# Differences between unipolar and bipolar I depression in the quantitative analysis of glutamic acid decarboxylase-immunoreactive neuropil

**DOI:** 10.1007/s00406-012-0315-x

**Published:** 2012-04-13

**Authors:** Tomasz Gos, Johann Steiner, Hendrik Bielau, Henrik Dobrowolny, Karoline Günther, Christian Mawrin, Maciej Krzyżanowski, Roman Hauser, Ralf Brisch, Hans-Gert Bernstein, Zbigniew Jankowski, Katharina Braun, Bernhard Bogerts

**Affiliations:** 1 Institute of Forensic Medicine, Medical University of Gdańsk, ul. Dębowa 23, 80-204 Gdańsk, Poland; 2 Department of Psychiatry, Otto-von-Guericke-University, Magdeburg, Germany; 3 Institute of Neuropathology, Otto-von-Guericke-University of Magdeburg, Magdeburg, Germany; 4 Department of Zoology, Developmental Neurobiology, Institute of Biology, Otto-von-Guericke-University, Magdeburg, Germany

**Keywords:** GAD 65/67, Postmortem, Depression, Unipolar–bipolar dichotomy

## Abstract

Alterations in GABAergic neurotransmission are assumed to play a crucial role in the pathophysiology of mood disorders. Glutamic acid decarboxylase (GAD) is the key enzyme in GABA synthesis. This study aimed to differentiate between unipolar and bipolar I depression using quantitative evaluation of GAD-immunoreactive (GAD-ir) neuropil in several brain regions known to be involved in the pathophysiology of mood disorders. Immunohistochemical staining of GAD 65/67 was performed in the orbitofrontal, anterior cingulate and dorsolateral prefrontal cortex (DLPFC), the entorhinal cortex, the hippocampal formation and the medial dorsal and lateral dorsal (LD) thalamic nuclei, with a quantitative densitometric analysis of GAD-ir neuropil. The study was performed on paraffin-embedded brains from 9 unipolar and 12 bipolar I depressed patients (8 and 6 suicidal patients, respectively) and 18 matched controls. In unipolar patients, compared with controls, only the increased relative density of GAD-ir neuropil in the right LD was different from the previous results in depressed suicides from the same cohort (Gos et al. in J Affect Disord 113:45–55, 2009). On the other hand, the left DLPFC was the only area where a significant decrease was observed, specific for bipolar I depression. Significant differences between both diagnostic groups were found in these regions. By revealing abnormalities in the relative density of GAD-ir neuropil in brain structures, our study suggests a diathesis of the GABAergic system in mood disorders, which may differentiate the pathophysiology of unipolar from that of bipolar I depression.

## Introduction

Abnormalities in the GABAergic system are assumed to play a crucial yet largely unknown role in the pathogenesis of mood disorders [3, 9, 41]. Gamma-aminobutyric acid (GABA) is the principal inhibitory neurotransmitter in the mature brain and plays an important role in synchronised neural network oscillations [17], synaptic plasticity [26] and neurogenesis [25]. The multitude of postmortem, clinical and preclinical studies predominantly suggests a hypothesis of low GABAergic activity in mood disorders (for recent reviews see references [3, 13, 30, 31]).

The quantitative neuropathological differences between major depressive disorder (MDD) and bipolar disorder (BD) have been addressed in neurohistological [7, 12] and molecular studies [15, 37, 40] on the GABAergic system. The pathological changes in markers related to this system, such as decreased parvalbumin mRNA in the dorsolateral prefrontal cortex (DLPFC) [37] and reduced density of glutamic acid decarboxylase 65/67-immunoreactive (GAD 65/67-ir) neurons in DLPFC and temporal cortical areas [7] specific for BD, differentiated this diagnostic entity from MDD. GAD is the rate-limiting enzyme involved in the conversion of glutamate to GABA with two isoforms, GAD 65 and GAD 67. Both of these functionally different isoforms are present in most GABA-containing neurones in the brain, but the transiently activated GAD 65 appears to be restricted to membranes and nerve endings, whereas the constitutively active GAD 67 is more widely distributed in cells [28, 42].

Despite the prevailing similarities, clinical studies accentuate some differences in symptom profiles and severity measures between unipolar and bipolar depression because of their important therapeutic implications (for reviews see references [16, 23, 38]). Similarities between them also predominate in neuroimaging studies (for a review see Ref. [36]) and according to the established consensus, a cross-sectional categorical distinction between unipolar and bipolar depression is currently impossible to make in clinical practice [16]. We hypothesised the presence of differences in the involvement of GABAergic neurotransmission in unipolar and bipolar I depression and have tested this hypothesis performing a quantitative evaluation of GAD 65/67-ir neuropil in several brain regions known to be involved in the pathophysiology of mood disorders.

## Materials and methods

### Subject characteristics

The demographic and clinical characteristics of the subjects are presented in Tables 1 and 2. The same cohort has previously been analysed according to the impact of suicide [21]. Briefly, postmortem brains from 21 depressed subjects with a clinical diagnosis of MDD (6 women and 3 men, ranging in age from 26 to 68 years) or BD subtype I (7 women and 5 men, ranging in age from 16 to 69 years), according to the *Diagnostic and Statistical Manual of Mental Disorders*, 4th Edition (DSM-IV), were obtained from the Magdeburg Brain Bank. Fourteen of these subjects had died by suicide (8 MDD and 6 BD patients) and the remaining seven had died from natural causes (1 MDD and 6 BD patients). The diagnosis of suicide was made by a forensic pathologist (T.G.). Control brains (C) were collected from 9 women and 9 men with an age range of 33 to 65 years, who had died from natural causes. The postmortem interval (PMI) ranged from 5 to 96 h for patients and from 19 to 72 h for the controls. A toxicology screen of blood and urine for ethanol, other substances of abuse, multiple antidepressant and antipsychotic drugs and their metabolites was performed at each medico-legal autopsy.

**Table 1:** Characteristics of subjects

	No./Sex/Age (years)	Psychiatric diagnosis (DSM-IV)	Cause of death	PMI (h)
Control group	1/M/50		MI	72
	2/M/47		CF	24
	3/F/52		Ovarian carcinoma	24
	4/M/47		Respiratory insufficiency	24
	5/F/64		Sepsis	24
	6/F/33		CF	72
	7/M/38		CF	19
	8/F/50		Dissecting aortic aneurysm	72
	9/M/64		HF	35
	10/F/48		PE	26
	11/M/56		HF	24
	12/F/65		HF	24
	13/F/30		PE	48
	14/M/63		HF	48
	15/F/64		HF	24
	16/F/38		PE	24
	17/M/54		RHF	24
	18/M/46		Lymphoma	24
MDD group	1/F/63	MDD (296.34)	PE	17
	2/M/42	MDD (296.33)	Hanging	5
	3/F/46	MDD (296.24)	Hanging	48
	4/F/53	MDD (296.33)	Hanging	46
	5/F/26	MDD (296.33)	Fall from the height	22
	6/F/68	MDD (296.33)	Hanging	96
	7/M/35	MDD (296.33)	Incision of radial artery	15
	8/M/36	MDD (296.34)	Hanging	42
	9/F/39	MDD (296.23)	Overdose of medication	48
BD group	10/F/62	BD-D (296.54)	PE	72
	11/M/39	BD-D (296.54)	CF	56
	12/M/69	BD-D (296.54)	PE	48
	13/F/52	BD-D (296.54)	PE	24
	14/F/65	BD-D (296.54)	HF	52
	15/M/44	BD-D (296.54)	HF	96
	16/F/53	BD-D (296.53)	Electric shock	47
	17/M/47	BD-D (296.53)	Stab wound	24
	18/F/16	BD-D (296.54)	Hanging	48
	19/F/46	BD-D (296.54)	Overdose of medication	4
	20/M/42	BD-D (296.54)	Hanging	17
	21/F/59	BD-D (296.54)	Overdose of medication	72

*M* male, *F* female, *PMI* postmortem interval, *MDD* major depressive disorder, *BD-D* bipolar I disorder depressed, *CF* coronary failure, *HF* heart failure, *MI* myocardial infarction, *PE* pulmonary embolism, *RHF* right heart failure

**Table 2:** Mean daily doses of psychotropic medication in the last 90 days of lifetime (benzodiazepines—in the last 28 days of lifetime)

Patient no.	Antidepressants (amitriptyline equivalents) mg	Neuroleptics (chlorpromazine equivalents) mg	Benzodiazepines (diazepam equivalents) mg	Lithium mg
1	50	0	0	0
2	0	0	0	0
3	124	109	0	0
4	0	0	0	0
5	0	0	0	0
6	0	0	0	0
7	0	0	0	0
8	0	0	0	0
9	93	0	3.1	560
10	0	110	17.6	0
11	0	221	0.8	740
12	0	0	6.8	0
13	0	0	0	0
14	93	117	3.9	0
15	0	0	0	0
16	67	0	0	0
17	20	0	0	0
18	0	0	0	0
19	133	327	3.3	558
20	95	47	18.3	565
21	112	140	10	0

The study was approved by the local ethics committee of the University of Magdeburg as being compliant with the Declaration of Helsinki of 1989 and the applicable EU and German laws. In accordance with the German autopsy laws, informed consent was obtained from the relatives of the deceased for autopsy and dissection of the brains and for use of clinical information for research purposes.

Antemortem DSM-IV diagnoses were obtained from psychological autopsies involving a careful review of clinical records and the performance of structured interviews with the physicians involved in the treatment and at least one person who either had lived with or had frequent contact with the subject before death. The DSM-IV Axis I diagnosis of MDD and BD subtype I was established in a consensus meeting by two independent psychiatrists (H.B. and J.S.). The same procedure was followed to exclude psychiatric disorders in controls. All BD patients were in a depressive episode at the moment of death. There was no current or lifetime psychoactive substance disorder history (abuse or dependence according to DSM-IV) in any of the subjects.

The mean doses of psychotropic medication in the last 90 days of life were established according to the clinical records, taking into account the equivalents of psychotropic medication present in the references [8, 10, 32, 33]. The majority of patients (12 of 21) had received psychotropic medication in this period. However, the medicated patients prevailed in the BD group (9 out of 12) compared with the MDD group (3 of 9).

Qualitative neuropathological changes due to neurodegenerative disorders (such as Alzheimer’s disease, Parkinson’s disease and Pick’s disease), tumours, inflammatory, vascular (microangiopathy, infarctions, lacunar infarctions and Binswanger’s disease) or traumatic processes were ruled out by an experienced neuropathologist (C.M.). The alterations suggestive of neurodegenerative disorders were excluded by immunostaining for beta-amyloid, hyperphosphorylated tau-protein and ubiquitin as well as by Gallyas silver stain.

### Tissue collection and preparation

After removal, brains were fixed in toto in 8 % phosphate-buffered formaldehyde for at least 2 months (pH = 7.0, *t* = 15–20 °C). Subsequently, after separation of the brainstem with the cerebellum, the hemispheres were divided by coronal cuts into three bi-hemispherical coronal blocks comprising the frontal lobe anterior to the genu of the corpus callosum (‘anterior’ block), the fronto-temporo-parietal lobe extending over the whole length of the corpus callosum (‘middle’ block) and the occipital lobe (‘posterior’ block). The ‘middle’ block included, in addition to the cortical areas, the thalamus and the hippocampal formation. The shrinkage of brain tissue during paraffin embedding was evaluated for each brain by calculating the ratio of identical distances between the opposite cortical gyri on the most rostral and the most caudal sections of the ‘middle’ block before and after embedding. The individual volume shrinkage factors (VSF) were calculated from the measured linear shrinkage factor (LSF) using the following formula: VSF = (LSF)^3^. The mean shrinkage factor was 2.25 for control brains, 2.27 for MDD patients and 2.13 for BD patients (a non-significant Kruskal–Wallis test with the three groups as independent variable). Two randomly selected coronal sections with a thickness of 20 μm per above-described ‘anterior’ block (at the level of the pregenual part of the anterior cingulate cortex) and two per ‘middle’ block (at the level of pes hippocampi) were immunohistochemically stained for GAD. Samples from all subject groups and all brain regions were included in every batch together with standard normalisation samples.

### Immunohistochemistry

**Fig. 1:**
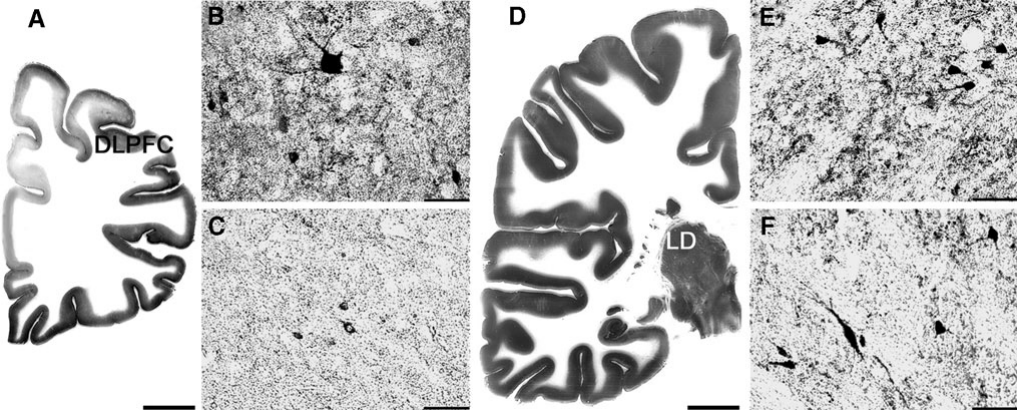
GAD 65/67 immunoreactivity in the left DLPFC (dorsolateral prefrontal cortex) and the right LD (lateral dorsal thalamic nucleus) of unipolar (**b** and **e**, respectively) and bipolar I (**c** and **f**, respectively) depressed patients; *scale bars* 50 μm. These regions of interest are shown at low magnification in pictures of GAD-immunostaining of a control case (**a** and **d**; *scale bars* 10 mm)

In order to immunolocalise GAD, a monoclonal antibody to GAD 65 and 67 in mice was used (Medical & Biological Laboratories Co., Wobrun, USA). The specificity of the antiserum was confirmed by the supplier by Western blotting and immunocytochemistry. After preincubation of the sections with methanol/H_2_O_2_ to depress endogenous peroxidases and repeated washing with phosphate-buffered saline, the primary GAD antibody was used at a 1:100 dilution for 72 h at 4 °C. Sections were then incubated with a biotinylated anti-mouse secondary antibody (Amersham Biosciences UK, Ltd., Little Chalford, UK) for the application of the avidin–biotin technique (Amersham). The chromogen 3,3′-diaminobenzidine (DAB) and ammonium nickel sulphate hexahydrate were used to visualise the reaction product. For the purposes of control, the primary antiserum was replaced either by a buffer or by normal serum and did not show any immunostaining in the investigated regions. Figure 1 shows the GAD-immunostaining pattern in the DLPFC and the lateral dorsal thalamic nucleus (LD).

### Quantification

A quantitative morphological analysis was performed in each of the selected sections as reported previously [21]. The relative density of GAD-ir neuropil (the quotient of the area of fibres and/or synaptic endings and total measuring field area, see below) of depressed patients and controls was measured in neocortical areas: prefrontal [DLPFC, orbitofrontal (OFC) and pregenual anterior cingulate] and temporal [parahippocampal gyrus containing the entorhinal cortex (EC)], in the hippocampal formation [dentate gyrus (DG) and the CA1 field of the hippocampus (CA1)] and in thalamic nuclei (medial dorsal [MD] and LD). For each neocortical area, three boxes in layer III and further three in layer V were randomly selected and scanned bilaterally by a video-equipped system (Olympus BX60 microscope equipped with a ColorView Soft Imaging System digital camera) onto the computer using a 20× objective. For the hippocampal complex, the same procedure was carried out in the pyramidal layer of CA1 and in the granular layer of DG. For LD and MD, three boxes were approached bilaterally. The number of evaluated boxes was established by the statistical analysis of preliminary data in which ten boxes per structure were evaluated bilaterally.

The relative GAD-ir neuropil area of the regions mentioned previously was determined using a computer application for densitometric image analysis, AnalySIS^®^ Auto Version 3.2 (Soft Imaging System GmbH, Münster, Germany). For the purpose of measuring, the area of immunostained structures the immunoreactive neuropil was visualised via adjustments of minimum and maximum grey levels of the nickel-enhanced DAB precipitate under visual control. The area of the marked immunoreactive fibres and/or endings was calculated and divided by the total area of the measuring field and thus demonstrated as the relative area of immunoreactive neuropil. The aim was to detect the difference in GABAergic innervation among the analysed groups according to the method described previously [21] rather than obtaining absolute values of the stained neuropil density.

The measurements were performed by one of the authors (K.G.) blinded to the diagnosis. In order to establish inter-rater (K.G., T.G.) and test–retest reliability, repeated measurements for 5 brains were carried out. Intraclass correlation analyses yielded correlation coefficients ranging from 0.90 to 1.00 in both inter-rater and test–retest reliability evaluation.

### Data analysis

As normal distribution (i.e. significant results of the Kolmogorov–Smirnoff test) was not given in all the analysed regions, non-parametric statistical procedures were used. Firstly, a Kruskal–Wallis analysis of the variance of ranks (*H* test) was performed using the diagnostic group as a 3-level independent variable (unipolar vs bipolar I depressed patients vs controls) and GAD-ir neuropil density, respectively. Secondly, the unadjusted two-way post hoc comparisons with the Mann–Whitney *U* test were carried out to detect between-group differences.

The same statistical procedures were employed to detect the possible differences between the groups according to the age at death, brain weight, postmortem delay and the time of fixation. The χ^2^-test was employed to detect the possible differences between them with respect to sex. The unadjusted two-way comparisons with the *U* test were employed to detect the possible differences between MDD and BD patients according to the age at the onset, duration of both the illness and depressive episode and psychotropic medication.

Spearman correlation coefficients were calculated to determine the impact of the above demographic, clinical and methodical variables, which might confound the results of dependent variable.

Generally, *P* values of <0.05 were accepted as statistically significant. When both the *H* test and triple post hoc comparisons with the *U* test were considered in combination, the *P* values were corrected for multiple comparisons in line with the Bonferroni–Holm–Shaffer procedure.

## Results

### Density of GAD-ir neuropil

The detailed results of group comparisons are shown in Table 3.

**Table 3:** Presentation of significant diagnostic group differences regarding GAD-immunoreactive neuropil relative density

ROI and group	GAD-ir neuropil density	H test *P*	Quotient of medians		
	Median (q1, q3; *n*)		MDD/C	BD/C	MDD/BD
CA1 left					
C	0.004 (0.001, 0.253; 18)	0.017	309**	4	88*
MDD	1.234 (0.723, 3.337; 9)				
BD	0.014 (0.002, 0.916; 12)				
DLPFC left III					
C	1.173 (0.016, 3.491; 18)	0.041	0.598	0.006*	100
MDD	0.702 (0.048, 2.899; 9)				
BD	0.007 (0.003, 0.029; 12)				
DLPFC left V					
C	0.552 (0.014, 5.346; 18)	0.015	2	0.013*	115**
MDD	0.806 (0.215, 3.598; 9)				
BD	0.007 (0.002, 0.018; 12)				
EC left III					
C	0.008 (0.003, 0.029; 18)	0.001	64**	40**	2
MDD	0.510 (0.167, 1.735; 9)				
BD	0.321 (0.050, 1.777; 12)				
EC left V					
C	0.011 (0.006, 0.035; 18)	0.016	59**	17	4
MDD	0.649 (0.416, 1.143; 9)				
BD	0.184 (0.012, 1.814; 12)				
EC right III					
C	0.012 (0.003, 0.069; 18)	0.009	46*	13*	4
MDD	0.548 (0.330, 1.482; 9)				
BD	0.152 (0.015, 1.275; 12)				
EC right V					
C	0.023 (0.005, 0.073; 18)	0.029	29**	5	6
MDD	0.662 (0.329, 2.895; 9)				
BD	0.103 (0.003, 1.303; 12)				
LD right					
C	0.237 (0.086, 0.513; 18)	0.028	4*	0.802	6*
MDD	1.048 (0.489, 1.434; 9)				
BD	0.190 (0.072, 0.399; 12)				

*ROI* region of interest, *GAD-ir* GAD-immunoreactive, *C* controls, *MDD* major depressive disorder, and *BD* bipolar I disorder depressed patients, *q1* and *q3* quartile 1 and 3,*n* number of cases, *CA1* CA1 field of hippocampus, *DLPFC* dorsolateral prefrontal cortex, *EC* entorhinal cortex, *LD* lateral dorsal thalamic nucleus; *III* layer III, and *V* layer V of pyramidal cells in neocortex; *H* test *P—H* test *P* values; * *P* < 0.05, ** *P* < 0.01 (*P* values corrected for multiple comparisons are related to the two-groups comparisons by post hoc *U* tests; values of the quotient of medians higher than 1 were rounded off to the whole numbers)

### Confounders

Variables that could influence GAD-ir neuropil density, such as PMI, time of fixation, shrinkage factor, sex, age at the time of death, brain weight, duration of both the illness and depressive episode, age at the onset of illness and psychiatric medication, were analysed.

The only significant difference existed in the mean duration of illness, which was significantly higher in BD versus MDD patients (17.3 vs. 4.2). The non-normal distribution of data excluded the implementation of analysis of covariance (ANCOVA). However, the duration of illness did not correlate with GAD-ir neuropil density in those regions of interest where significant differences in the investigated parameter between the compared diagnostic groups were found. A positive correlation between the confounding and dependent variables was found in the left OFC in both of the investigated cortical layers in BD patients only (*r* = 0.71, *P* = 0.047).

There were no significant differences in the mean doses of psychotropic medication between the compared groups of patients. However, there were significant, mostly negative correlations between GAD-ir neuropil density and the mean doses of psychotropic medication, predominantly in BD patients (Table 4).

**Table 4:** The significant correlations found between psychotropic medication and GAD-immunoreactive neuropil relative density in regions of interest of unipolar (MDD) and bipolar I (BD) depressed patients (antidepressants, neuroleptics and lithium—in the last 90 days, benzodiazepines—in the last 28 days of lifetime)

ROI	AD		NL	BDZ	Lithium
	MDD	BD	BD	BD	BD
ACC left V	0.89*				
DG left			−0.85*		
EC left III		0.82*			
EC right V			−0.94*		
MD left	−0.89*		−0.76*		
OFC left III				0.72*	
OFC right III			−0.73*		
OFC right V					−0.76*

*ROI* region of interest, *AD* antidepressants, *NL* neuroleptics, *BDZ* benzodiazepines, *ACC* anterior cingulate cortex, *DG* dentate gyrus, *EC* entorhinal cortex, *MD* medial dorsal thalamic nucleus, *OFC* orbitofrontal cortex, *III* layer III, and *V* layer V of pyramidal cells in neocortex; the values of correlation coefficients *r* are shown; * *P* < 0.05

## Discussion

### Diagnostic issues

Compared with healthy controls, the increased density of GAD-ir neuropil prevailed in the evaluated cerebral regions of depressed patients, being accentuated in the EC and the hippocampus, predominantly in MDD (Table 3). MDD patients had increased values of this parameter compared with BD patients and controls in the left CA1 and the right LD. The left DLPFC was the only area where significantly decreased GAD-ir neuropil density was found in BD compared with MDD patients and controls.

We have recently revealed in a series of publications that processes leading to suicide have an outstanding impact on the observed neurohistological abnormalities independent from the main Axis I diagnosis [18–22, 39]. This impact was also accentuated in our previous quantitative study on GAD-ir neuropil in the same cohort [21], where we found an increase in its density specific for depressed suicidal patients from the MDD and BD diagnostic groups. Therefore, the impact of suicide should be considered in the interpretation of our current results.

Because suicidal patients prevailed in the MDD group (8 out of 9), the increased GAD-ir neuropil density observed in this group could be related to suicide in regions where the increase had previously been found [21], such as in the left CA1. The right LD was the only region in MDD patients where the currently observed increase did not overlap with previous findings in the suicidal group. Therefore, only the increased parameter in this structure involved in separate aspects of episodic memory [1, 11, 35] should be considered as being specific for unipolar depression (Fig. 1).

On the other hand, the BD group was balanced in terms of suicidal versus non-suicidal patients. Therefore, the decreased GAD-ir neuropil density currently observed in the left DLPFC seems to be specific for bipolar I depression (Fig. 1) when considering an opposite effect previously found in suicide [21]. A recent immunoblotting study [27] revealed that the amount of GAD 65 specific for axonal endings was unchanged in DLPFC in both medicated and medication-free MDD patients, which is consistent with our interpretation of the present findings (Table 3).

The effect we observed was consistent with other data obtained in the PFC in both BD and schizophrenia [2, 5, 24, 40, 43]. Moreover, in line with the study on GAD 65 and GAD 67 gene expression in elderly schizophrenics [14], we found a positive correlation between the duration of illness and GAD-ir neuropil density in BD patients in one of the investigated PFC regions (namely in the left OFC; *r* = 0.71, *P* = 0.047). Therefore, both ours and the cited results for PFC support similarities between schizophrenia and BD.

### Influence of psychotropic medication

We demonstrated some significant correlations, predominantly negative, between the relative density of GAD-ir neuropil and the mean doses of psychotropic medication (Table 4). However, the correlations found in the MDD group are not very meaningful because only three MDD patients received antidepressants. On the other hand, in the BD group, correlations prevailed in areas where no significant differences in GAD-ir neuropil density were observed (5 out of 7). Moreover, the number of treated BD patients did not exceed 7 (out of 12) in each of the analysed groups of psychotropic agents (Table 2). Therefore, it cannot be conclusively stated whether medication did in fact confound our results.

Other human and concurrent preclinical studies have suggested that antidepressants might up-regulate GAD [6, 7, 27, 34]. In experimental conditions, antidepressants may also down-regulate the level of GABA in cortical areas [29]. Therefore, in the light of both previous [21] and present findings, antidepressant medication might exert an effect that possibly counteracts the abnormality in the GABAergic system. However, the putatively positive effect of pharmacotherapy is regionally differentiated and is absent in the DLPFC and LD (Table 4).

## Limitations

A major limitation of our study is the relatively small number of cases. For future studies, the numbers of cases from both diagnostic groups should be increased and subgroups of remitted MDD and BD I patients should be included in order to differentiate between trait and state conditions of the GABAergic system in uni- and bipolar depression. A further limitation of this study is the lack of data on lifetime drug exposure, as we could only collect data on psychotropic medication covering the last 3 months before death. Moreover, especially in studies investigating mood disorders, the heterogeneity of the results may likely be caused by clinical sample variation. The available well-documented clinical files do not suggest that some of our MDD patients were actually BD ones. However, we cannot unequivocally exclude this doubt existing in view of the current criticism on the MDD concept [4]. The implementation of a monoclonal antibody to both isoforms of GAD should be considered as another limiting factor in the interpretation of our results and in the discussion with the overwhelming majority of previous studies which have applied isoform-specific antibodies.

## Conclusion

When considering previous results regarding the influence of suicide, an increase in the relative density of GAD-ir neuropil in the right LD seems to be specific for MDD, whereas its decrease observed in the left DLPFC may be pathognomonic of bipolar I depression. By revealing a diagnosis- and brain region-specific pattern of abnormalities in the GAD-ir, our study suggests a diathesis of the GABAergic system, which may differentiate the pathophysiology of unipolar from that of bipolar I depression. The suggested diathesis of the GABAergic system in depression may be partially regulated by psychotropic medication.
